# Grounded theory - a lens to understanding clinical reasoning

**DOI:** 10.15694/mep.2017.000002

**Published:** 2017-01-05

**Authors:** Paul Welch, David Plummer, Louise Young, Frances Quirk, Sarah Larkins, Rebecca Evans, Tarun Sen Gupta

**Affiliations:** 1 James Cook University; 2 Griffiths University; 3 Barwon Health and Deakin University

**Keywords:** clinical reasoning, teaching clinical reasoning, researching clinical reasoning, clinical interviewing, grounded theory

## Abstract

This article was migrated. The article was marked as recommended.

Clinical reasoning is fundamental to medical education and clinical practice ( Schmidt & Mamede, 2015). Despite its centrality, clinical reasoning is often regarded as difficult to conceptualise and teach ( Charlin, 2012; Pinnock & Welch, 2014). The pivotal role of clinical reasoning constitutes a compelling case for better understanding, more efficient teaching and practice that is more systematic and evidence-based. Clinical reasoning has been regarded as an art rather than a science ( Braude, 2012) and has attracted less research effort than befits its important function. The authors examined the suitability of grounded theory methodology to provide a more complete understanding of the clinical reasoning process. Grounded theory and clinical reasoning are processes which both qualitatively evaluate and analyse information from an interview subject as well as additional sources and arrive at a robust, defensible theory to explain their findings. Grounded theory offers considerable utility for (i) understanding and modelling clinical reasoning, (ii) researching clinical reasoning and (iii) as a heuristic for teaching clinical reasoning skills. This paper explores the parallels between grounded theory methodology and clinical reasoning, as well as the suitability of grounded theory as a framework for informing and transforming our understanding of clinical reasoning.

## Background

Evidence-based practice is central to modern health care. In medicine, evidence has often been taken to mean the knowledge gained through the application of quantitative scientific methods, such as laboratory experiments and clinical trials. Regardless of how robust the scientific data or how precise the technology used, clinical medicine remains an interpretative practice ( Montgomery, 2005). Clinical reasoning is the cognitive process of evaluating and managing a patient’s problems ( Barrows & Tamblyn, 1980) and makes practical use of empirical medical knowledge and evidence. For clinicians, it is particularly significant that qualitative inquiry is regularly used to make critical clinical decisions. This is despite there being an entrenched scepticism about qualitative methods elsewhere in the medical sciences ( Malterud, 2001; Sofaer, 2002). We suggest that for evidence-based healthcare to be most effective, there is a pressing need for a more systematic understanding of the handling and analysis of the qualitative information generated during each clinical interaction.

Despite appeals for medicine to be ‘evidence-based’ and ‘scientific’, clinical reasoning does not conform to the conventional criteria for a scientific methodology. It uses a sample size of one (the patient), employs individual interviews to gather information, analyses and interprets imaging information qualitatively, qualitatively interprets objective quantitative laboratory results and takes an iterative approach to arrive at the final diagnosis. Furthermore, clinical reasoning is often a shared cognitive process taking place in a busy and time-pressured environment, involving conversations between the patient, clinicians and often relatives. It is suggested that an appropriate model for understanding clinical evaluation needs to embrace and reflect these characteristics. A broader look at robust qualitative research methods that meet the demands of academic peer review may well have utility to better understand clinical reasoning.

Advancing our understanding and modelling of clinical reasoning should enable us to teach it better. Clinical reasoning research to date has progressed along two primary axes: Information processing theories and situativity theories ( Durning & Artino, 2011). Conceptual complexity, case-to-case irregularity and ill-structured knowledge domains in medicine pose significant problems for traditional learning theories ( Coulson, Feltovich, & Spiro, 1997). In the late 1980s, Spiro and colleagues developed *Cognitive Flexibility Theory* as a means of refining traditional learning theories to accommodate advanced knowledge acquisition in ill-structured domains like medicine ( Spiro, Coulson, Feltovich, & Anderson, 1988). The theory challenges and seeks to remediate some of the common errors of learners, for example, the tendency to oversimplify complex concepts and an overreliance on a single basis for mental representations ( Spiro et al., 1988). Understanding this complexity and case-by-case variability led Schmidt to identify distinct phases the novice passes through on their way to developing expertise in clinical reasoning ( Schmidt, Norman, & Boshuizen, 1990). These phases help explain the need for the learner to understand, assimilate and produce their own mental schema linking and storing information for future retrieval and use in the clinical situation. This stored data is later used to enable pattern recognition, which is increasingly used in the clinical reasoning process as experience and expertise develop. Each clinical case is slightly different and requires a reasoning process tailored to the case ( Mandin, Jones, Woloshuk, & Harasym, 1997).

This paper proposes using grounded theory as a framework for understanding and explaining clinical reasoning and for harnessing the data analysis methodology used by grounded theory as a means of coaching clinical reasoning ( Ericsson, 2004; Pinnock et al, 2014). Glaser and Strauss first described grounded theory in their seminal 1967 book *The discovery of grounded theory* ( Glaser & Strauss, 1967). Since then, grounded theory has been extensively deployed and peer reviewed as a data gathering and analysis tool in qualitative research ( Charmaz, 2014); evolved into an accepted systematic approach that copes well with relatively small sample sizes and complex systems; and been adapted to allow for the incorporation of evidence in the form of documents, observations and artefacts ( Charmaz, 2016; Layder, 1993). This is relevant in the medical context where additional information such as laboratory test results and imaging data help to shape the decision-making process. In recent years grounded theory has become accepted as a useful methodology in medical education research ( Watling & Lingard, 2012). The complexity of clinical reasoning entails information gathering, storage, retrieval and use highlighting the need to identify a framework to coach clinical reasoning that accommodates these features ( Mandin et al., 1997).

## Clinical reasoning viewed as a qualitative researcher

Qualitative research methods are widely accepted by the academic research community and regularly satisfy the rigours of peer review ( Yamazaki et al., 2009). The close parallels between the process of clinical reasoning and that used in grounded theory led us to hypothesise that grounded theory might offer an invaluable explanatory model for clinical reasoning. Charmaz’s constructivist grounded theory ‘brings to the fore the notion of the researcher as author’ ( Mills, Bonner, & Francis, 2006). This approach resonates strongly with the clinical reasoning process where the clinician gathers and interprets this clinical information. Below we expand on how this grounded theory approach is relevant to the clinical reasoning process. Similarities between grounded theory and clinical reasoning will be discussed by considering: i. Prior knowledge and experience; ii. Sampling and data collection; iii. Data analysis procedures and iv. Data logic.

**Table 1.**
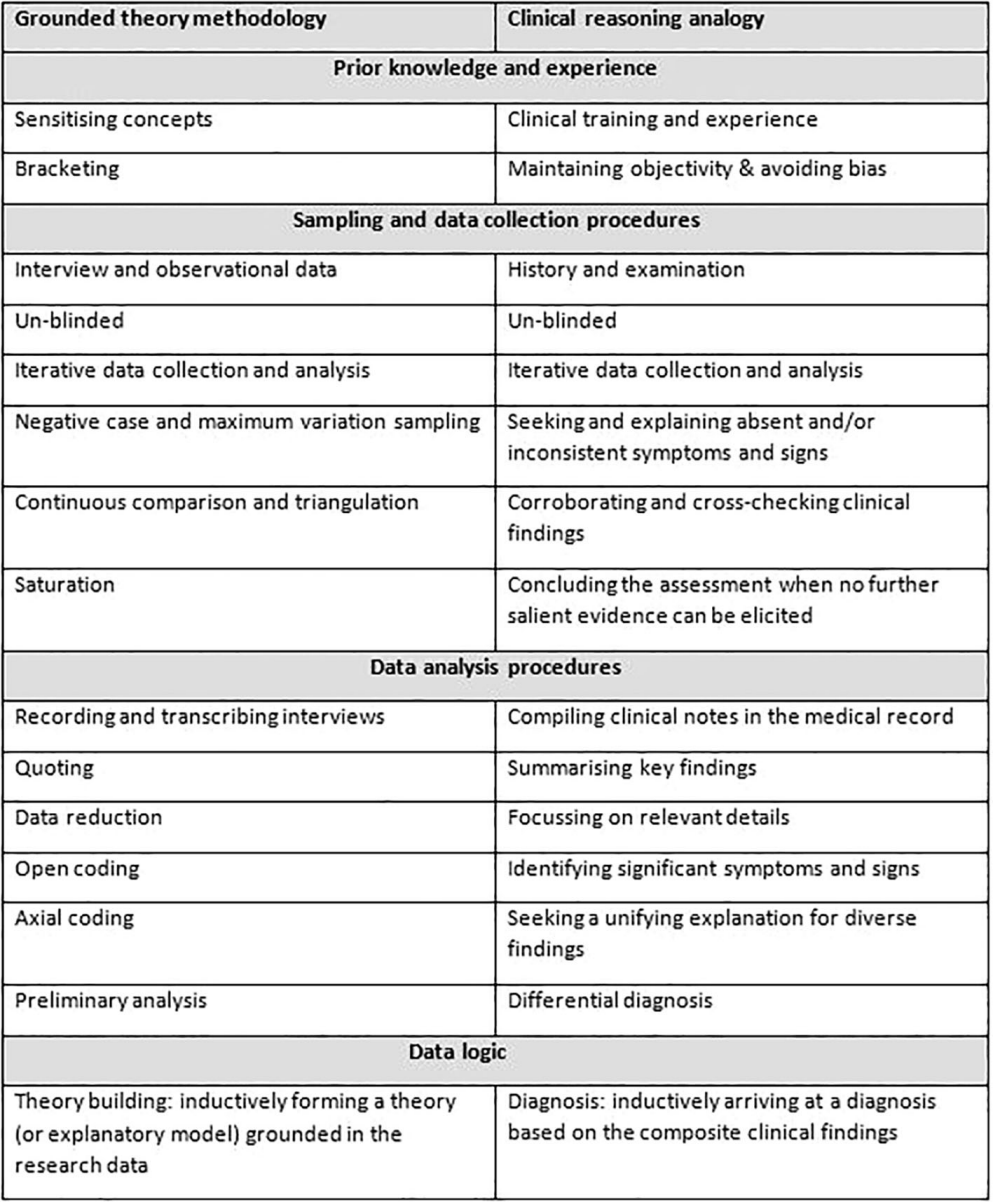
Comparison of grounded theory and clinical reasoning

## (i) Prior knowledge and experience

As with every research project, each clinical interaction is informed by prior knowledge, experience and insights. These pre-conditions, known to researchers as ‘sensitising concepts’, represent the insights the researcher brings to the analysis and provide a necessary basis for launching the inquiry ( Bryant & Charmaz, 2007). Implicit in the idea of ‘sensitising concepts’ is recognition that the researcher is not a blank slate. In a similar way, the clinician does not, and should not, come to a new case without prior training and knowledge.

For the researcher, the challenge is to leverage these insights for the benefit of the inquiry without unduly pre-empting the outcomes before the evidence is fully delineated. For researchers, this leads to a counterbalancing concept known as ‘bracketing’ ( Bryant & Charmaz, 2007; Creswell, 2013; Janesick, 2000; Kvale & Brinkmann, 2009). Bracketing acknowledges the need to hold pre-existing knowledge in abeyance in order to take a fresh look at the data with the aim of being open to alternative, possibly better, explanations. So for the qualitative researcher, there is a recognition of the importance of maintaining a balance between using prior knowledge to inform the investigation, and preventing it from prejudicing the current inquiry by pre-empting the outcome. Clinicians think of this balance as maintaining ‘objectivity’ and avoiding ‘biases’ to prevent the error of premature closure ( Scott, 2009). In the clinical context, each case is subtly different, requiring both inductive and deductive approaches to careful information gathering which is targeted at making a diagnostic or management decision. Yet in order to reach a diagnosis, the clinical process seeks to discover the features each unique case has in common with other cases and the pre-existing evidence-base. Matching the patient’s unfolding clinical story against the clinician’s store of previously stored cases is a deductive process. Whilst carefully gathering and synthesising information the clinician is mentally searching for a name or description to assign to the sum of the patient’s salient presenting complaints to arrive at a diagnosis.

## (ii) Sampling and data collection

The ultimate aim of a grounded theory study is to collect and analyse data systematically in order to develop a theoretical model that explains the phenomenon under study ( Glaser & Strauss, 1967; Layder, 1993). A diagnosis that arises from a clinical presentation is very similar and involves a theory about the presenting patient that is grounded in the data obtained during the clinical inquiry.

A striking parallel between the clinical assessment and grounded theory is that both revolve around the conduct and analysis of interviews and are supplemented by information gathering. Like a clinician, a grounded theory researcher would typically start an interview with broad, non-specific questioning and encourage the participant to relate their account in their own words ( Kvale & Brinkmann, 2009). Possible explanations start to emerge as the interview unfolds and the inquiry becomes increasingly focused until it is judged that sufficient data has been collected to draw the investigation to a close. Both the clinical interview and the grounded theory interview follow very similar formats.

## (iii) Data analysis procedures

Sampling decisions are a central issue for rigorous interview-based qualitative studies ( Bryant & Charmaz, 2007). Likewise, sampling, in terms of choosing suitable interview probes, is important for clinical reasoning. The clinical assessment can be led dangerously astray by poorly chosen questions as well as imaging or laboratory findings that distract from an underlying diagnosis. To manage these issues, the grounded theory researcher takes a procedural approach to sampling, and this strategy offers valuable clues for the clinical assessment.

Grounded theory research uses a form of *purposive sampling*, known as *theoretical sampling* ( K. Charmaz, 2000; Creswell, 2013; Merkens, 2004). As the name suggests, sampling is conducted with a particular *purpose* in mind, in this case, to build a *theory* to explain what is being observed. Like a diagnosis, many grounded theories are simply modest models that help to explain the accumulating evidence. In that sense, a grounded theory shares many similarities with a diagnosis, which can be considered to be a grounded theory that seeks to provide a unifying explanation for the patient’s presenting complaint.

There are two levels of data sampling that take place in qualitative research. The first consists of the selection of research participants and the second consists of the choice of probes used in individual interviews. In terms of sampling which participants to interview, the grounded theory researcher seeks key informants who are in a position to share information and experiences that shed light on the phenomenon being studied. In contrast, the history taking and the tests a clinician might order are pre-determined by the manner in which the patient presents to the clinic. In the case of sampling data from individual participants during an interview, the grounded theorist researcher is guided by the need to develop a coherent explanatory model or theory (thus the term *theoretical sampling*). Likewise, for the clinician, their questions, physical examination, imaging and tests ordered are guided by the need to devise a unifying model that explains the various clinical findings so as to arrive at a diagnosis or management plan. These sampling decisions further highlight the similarities between grounded theory and the clinical reasoning process.

Neither clinical reasoning nor grounded theory analysis are blinded processes, nor should they be - randomisation has no role here. Interviews do not consist of a random selection of questions and statements but are systematic and sequential as earlier revelations will guide subsequent questioning. Both qualitative research methods and clinical reasoning take an iterative approach to data collection and analysis that is designed to accumulate evidence, test possibilities, seek out alternatives and construct explanations. Similarly, for clinical data gathering, it is standard practice in grounded theory research to revise the sampling approach and to adjust the interview guide in the light of the findings that emerge as the study unfolds. In this way, both the clinical reasoning process and qualitative research study progressively focus in on an emerging explanation.

**Figure 1.**
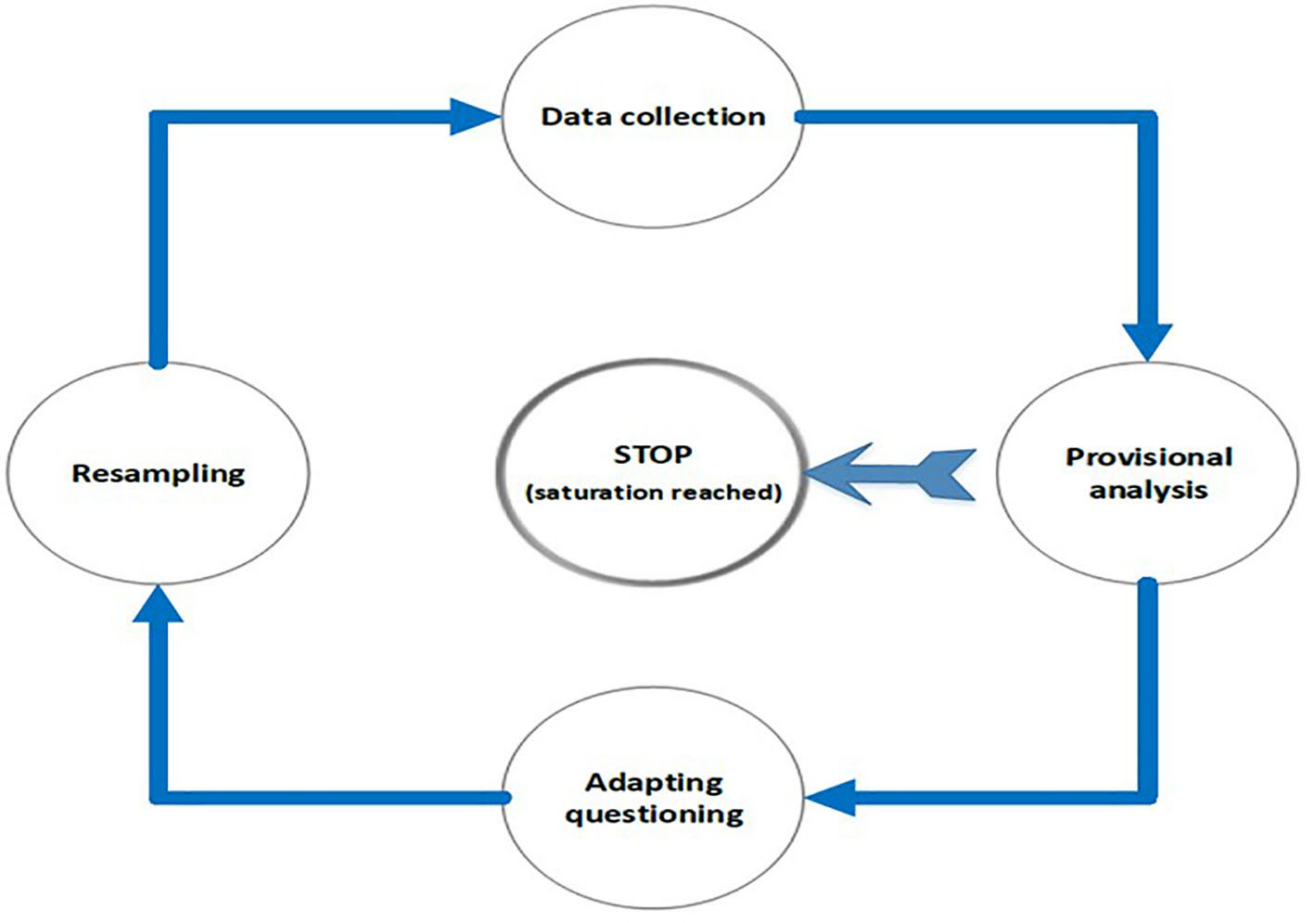
Clinical assessment and reasoning is iterative, un-blinded and inductive

Qualitative research methods, and in particular grounded theory, come to the fore when exploring the unknown, when analysing complex systems, and when handling relatively small sample sizes. Each of these characteristics also applies to clinical reasoning for an unknown diagnosis, complex pathophysiological systems, and a sample size of one. In order to adapt to these requirements, qualitative research has developed a specialised approach to determining the sample size known as *saturation* ( Bryant & Charmaz, 2007; Creswell, 2013). This approach specifies that sampling should continue until the data become repetitive, and no new and/or variant data can be discovered. Thus the sample size only becomes finalised while data is being collected ( Figure 1). A similar situation prevails for clinical assessment where the data gathering is drawn to a close when additional clinical details no longer add further clarity to the clinical picture that has emerged.

A further characteristic of purposive sampling, known as variant case or maximum variation sampling ( Creswell, 2013), also offers important insights for clinical reasoning process. Variant case sampling entails seeking out and examining cases that vary from what has been observed, in order to make sure that the emerging theory is sufficiently robust and up to the task of explaining variant findings. Collecting a rich variety of accounts helps to elaborate a more robust and complete explanatory theory, as does the confirmation of one finding by another. This corroboration between findings is known as *triangulation* in qualitative research ( Creswell, 2013).

An extreme version of variant case sampling is negative case sampling ( Bryant & Charmaz, 2007). Here too, the emerging explanation must be able to explain why a characteristic is missing (negative case) when the explanation developed so far suggests it should be present. Identifying and analysing a variant should lead to the theory either being modified to take it into account or abandoned in favour of a better explanation. In this sense, qualitative research adopts a different approach from conventional scientific method, which may tend to dismiss anomalous data as statistical outliers. Grounded theory researchers and experts in clinical reasoning typically seek out and pay careful attention to ‘outliers’ because of their importance as indicators of whether the emerging explanation is adequate or not. If a clinician ignores ‘outlier’ information that seems not to fit the clinical picture it may have disastrous consequences leading to an entirely wrong diagnosis or management plan. The clinical reasoning literature emphasises the need for experts not to ignore ‘outlier’ information that does not appear to fit the clinical picture, but to continue to search for a diagnosis that accommodates all of the patient’s clinical data ( Marcum, 2012).

Following consideration of procedures related to grounded theory research and the steps in clinical reasoning comes the interpretation of data.

Analysis and interpretation of data in grounded theory research and how this relates to clinical reasoning is the next similarity.

## (iv) Data logic

At the core of grounded theory research is the analytical process. Unlike conventional scientific methods, which are sequential, controlled and blinded, the analytical process in grounded theory is iterative, un-blinded and takes place concurrently with data collection. In this regard, grounded theory research is very similar to the clinical reasoning process.

Analysis of grounded theory research interviews takes place at a number of points in the research process. First, constant analysis occurs *during the interview* and provides a basis for further (iterative) questioning as the interview unfolds. Second, there is preliminary analysis *after each interview.* At this stage, the analysis aims to: (a) review the interview for clues that will contribute to the progressive development of an explanatory model; (b) explore the material for new and unexpected possibilities and to revise the interview guide accordingly to explore these in more detail in subsequent interviews; and (c) to progressively revise the sampling approach using theoretical, maximum variation and negative case sampling in order to fill knowledge gaps and test alternative explanations. Third, there is a *formal summative analysis* which ultimately leads to the generation of an explanatory theory grounded in the data (a grounded theory).

Unlike clinical interviews, research interviews are typically audio-recorded and transcribed verbatim. The analysis is based on the evidence contained in these transcripts. The first step is to identify statements that appear to offer significant insights into the issues being studied. Deciding which statements are significant is based on their relevance to the research question, prior sensitising concepts, and preliminary analysis during interviews. This step is commonly known as ‘quoting’ ( Kvale & Brinkmann, 2009). Quoting serves a dual function of drawing out significant evidence in the form of statements made by participants (‘quotes’) while filtering out superfluous material and assisting with data reduction. Extracting notable quotes and filtering out superfluous details offers a systematic way of focussing and summarising interview data which in many qualitative projects can be voluminous. A similar result is achieved in the way clinical notes are written, which separate out and summarise pertinent data with the aim of formulating a diagnosis.

Once quotes are identified by the qualitative researcher, the next step is to code them see Saldana for a full exposition ( Saldana, 2013). Coding is a complex process which assigns one or more tag words or phrases to each quote. These tags have two primary functions: first, as a *label* that summarises a feature of a quote that sheds light on the research question (known as *open coding*); and second, to *classify* the quote and thus group it with other evidence to highlight the common ground and to reveal patterns and commonalities that help to explain the findings (known as *axial coding* in qualitative methods). Ultimately these thematic groupings of related codes can be used as the basis for postulating a coherent explanatory model that draws the various pieces of evidence together into a grounded theory. An analogous situation in a clinical setting would be a history of deep central discomfort (code: chest pain) that when related to other findings (axial coding) such as age, sex, smoking, family history, radiating pain down the arm or vomiting blood, could lead to the clinician theorising (a grounded theory) that the person is suffering a heart attack or gastric ulcer respectively.

## Summary

In summary, a number of simultaneous processes take place during a grounded theory interview or clinical reasoning ( Table 1). The overarching elements which aim to build a robust explanation include:

1. Progressive funnelling of questioning from broad open-ended questions towards a final clinical outcome (a process known as *continuous comparative* or *iterative analysis*)
2. Incorporation of clinical evidence in the form of observations, physical examination findings and clinical documentation (analogous to Layder’s grounded theory adaptations that introduce evidence from observations, documents and physical artefacts ( Layder, 1993))
3. Constant, un-blinded, comparative analysis applied to each new piece of clinical data to ensure that the resulting explanation is coherent and corroborated by as many other finding as possible (analogous to *triangulation*).
4. Actively seeking and explaining variant and negative findings because of their potential value as ‘flags’ for problems with the diagnosis, which may need revising (analogous to *maximum variation* and *negative case analysis*).
5. Drawing the clinical assessment to a close when no new data is forthcoming (known as *saturation*).
6. Arriving at a diagnosis grounded in the clinical findings (known as a *grounded theory* in grounded theory research).

## Conclusion

Clinical reasoning is a complicated process, and despite being the subject of an extensive body of work our understanding of it is far from complete. The primary approach to the clinical reasoning process is qualitative being based on the clinical history, physical examination and additional imaging and laboratory results. Due to these similarities, we turned to disciplines that have an established track record of using qualitative methods to shed further light on clinical reasoning. The clinical reasoning process shares many similarities with grounded theory including strong methodological similarities. Grounded theory is a valuable framework (i) for understanding and modelling clinical assessment and reasoning; (ii) for researching clinical reasoning; and (iii) for coaching clinical reasoning in the working clinical environment.

## Take Home Messages

• Clinical medicine is an interpretive practice not a precise science.
• Clinical reasoning is vital to medical practice, but difficult to conceptualise and teach.
• Grounded theory provides a robust methodology to better understand the clinical reasoning process.

## Notes On Contributors

**Paul Welch** - PhD candidate in the College of Medicine and Dentistry, James Cook University, Queensland

**Prof David Plummer** - Professor of Population Health, Griffiths University, Queensland

**A/Prof Louise Young** - Associate Professor of Rural Medical Education, James Cook University, Queensland

**Prof Frances Quirk** - Director of Research, Barwon Health, Victoria

**Prof Sarah Larkins** - Associate Dean, Research in the College of Medicine and Dentistry, James Cook University, Queensland

**Dr Rebecca Evans** - Lecturer in the College of Medicine and Dentistry, James Cook University, Queensland

**Prof Tarun Sen Gupta** - Professor of Medical Education, James Cook University, Queensland

## References

[ref1] Barrows H. S. & Tamblyn R. M. (1980). Problem-based learning: An approach to medical education. Springer Publishing Company.

[ref2] Braude H. D. (2012). Intuition in medicine - a philosphical defence of clinical reasoning. Chicago, Il: University of Chicago Press. 10.7208/chicago/9780226071688.001.0001

[ref3] Bryant A. & Charmaz K. (2007). The SAGE Handbook of grounded theory. London: Sage. 10.4135/9781848607941

[ref4] Charlin B. (2012). Clinical reasoning processes: unravelling complexity through graphical representation. Medical Education. 46,454–463. 10.1111/j.1365-2923.2012.04242.x 22515753

[ref5] Charmaz.(2014). Constructing grounded theory.( Seaman J. Ed. 2nd ed.). London: Sage.

[ref6] Charmaz.(2016). The power of constructivist grounded theory for critical inquiry. Qualitative Inquiry. 10.1177/1077800416657105

[ref7] Charmaz K. (2000). Grounded theory. Objectivist and constructivist models Handbook of qualitative research.( 2nd ed.). Thousand Oaks, Ca: Sage.

[ref8] Coulson R. L. Feltovich P. J. & Spiro R. J. (1997). Cognitive flexibility in medicine: An application to the recognition and understanding of hypertension. Advances in Health Sciences Education. 2(2),141–161. 10.1023/A:1009780229455 12386405

[ref9] Creswell J. W. (Ed.) (2013). Qualitative inquiry and research design.( 4 ed.). Thousand Oaks, CA: Sage.

[ref10] Durning S. J. & Artino A. R. (2011). Situativity theory: A perspective on how participants and the environment can interact: AMEE Guide no. 52. Medical Teacher. 33(3),188–199. 10.3109/0142159X.2011.550965 21345059

[ref11] Ericsson K. A. (2004). Deliberate practice and the acquisition and maintenance of expert performance in medicine and related domains. Academic Medicine. 79(10 SUPPL.),S70–S81. 10.1097/00001888-200410001-00022 15383395

[ref12] Glaser B. & Strauss A. (1967). The Discovery of Grounded Theory: Strategies for Qualitative Research. Chicago: Aldine Publishing Company.

[ref13] Janesick V. (2000). The choreography of qualitative research design.( Denzin N. K. & Lincoln Y. S. Eds. 2nd ed.). Thousand Oaks, Ca: Sage.

[ref14] Kvale S. & Brinkmann S. (2009). Interviews: learning the craft of qualitative research interviewing. Thousand Oaks, Ca: Sage.

[ref15] Layder D. (1993). New strategies in social research: An introduction and guide. London: Polity.

[ref16] Malterud K. (2001). Qualitative research: Standards, challenges, and guidelines. Lancet. 358(9280),483–488. 10.1016/S0140-6736(01)05627-6 11513933

[ref17] Mandin H. Jones A. Woloshuk W. & Harasym P. (1997). Helping students learn to think like experts when solving clinical problems. Academic Medicine. 72(3),173–179. 10.1097/00001888-199703000-00009 9075420

[ref18] Marcum J. A. (2012). An integrated model of clinical reasoning: Dual-process theory of cognition and metacognition. Journal of Evaluation in Clinical Practice. 18(5),954–961. 10.1111/j.1365-2753.2012.01900.x 22994991

[ref19] Merkens H. (2004). Selection procedures, sampling, case construction. Thosand Oaks, Ca: Sage.

[ref20] Mills J. Bonner A. & Francis K. (2006). The development of constructivist grounded theory. International Journal of Qualitative Methods. 5(1),25–35.

[ref21] Montgomery K. (2005). How doctors think: Clinical judgment and the practice of medicine. Oxford University Press.

[ref22] Pinnock R. & Welch P. (2014). Learning clinical reasoning. J Paediatr Child Health. 50(4),253–257. 10.1111/jpc.12455 24372846

[ref23] Saldana J. (2013). The coding manual for qualitative researchers.( 2nd ed.). London: Sage.

[ref24] Schmidt H. G. & Mamede S. (2015). How to improve the teaching of clinical reasoning: a narrative review and a proposal. Medical Education. 49(10),961–973. 10.1111/medu.12775 26383068

[ref25] Schmidt H. G. Norman G. R. & Boshuizen H. P. (1990). A cognitive perspective on medical expertise: theory and implication. Academic Medicine. 65(10),611–621. 10.1097/00001888-199010000-00001 2261032

[ref26] Scott I. A. (2009). Errors in clinical reasoning: causes and remedial strategies. BMJ. 338,b1860. 10.1136/bmj.b1860 19505957

[ref27] Sofaer S. (2002). Qualitative research methods. International Journal for Quality in Health Care. 14(4),329–336. 10.1093/intqhc/14.4.329 12201192

[ref28] Spiro R. J. Coulson R. L. Feltovich P. J. & Anderson D. K. (1988). Cognitive Flexibility Theory: Advanced Knowledge Acquisition in ill-structured domains Tenth Annual Conference of the Cognitive Science Society Proceedings. 1988pp.640–653

[ref29] Watling C. J. & Lingard L. (2012). Grounded theory in medical education research: AMEE Guide No. 70. Medical Teacher. 34(10),850–861. 10.3109/0142159X.2012.704439 22913519

[ref30] Yamazaki H. Slingsby B. T. Takahashi M. Hayashi Y. Sugimori H. & Nakayama T. (2009). Characteristics of qualitative studies in influential journals of general medicine: a critical review. Bioscience trends. 3(6). 20103848

