# Supplementary material for: Grounded theory - a lens to understanding clinical reasoning
Source: MedEdPublish (2016). 2017 Jan 5;6:2. [Version 1] doi: 10.15694/mep.2017.000002 (PMC10885213; doi:10.15694/mep.2017.000002)
Supplement: Peer review history

# Reviewer report: Reviewer response for version 1

**DOI:** 10.21956/mep.19413.r28496

**Published:** 2017-01-07

**Authors:** Ken Masters

**Affiliations:** 1 Sultan Qaboos University

This review has been migrated. The reviewer awarded 4 stars out of 5 I am not sure if the system works entirely, but the authors are certainly to be commended on a rather brave yoking of seemingly two disparate ideas, and I also believe that the paper could lead so some fascinating discussions. Perhaps the biggest area of contention is the sample size. True grounded theory is ideal for a small sample size, but I have never seen it applied to a sample of one. Indeed, the idea of saturation is not merely that no further data should be gathered, but that no further interview subjects (or focus group subjects) need to be consulted. In the paper, the authors appear to take the single patient as the entire population (not the sample), and then use the symptoms, signs, etc., as the individual items of the sample. Brave, but tricky. (Also, the use of variant/variation sampling might not be appropriate here, as the equivalent would be a GT study that then seeks out entirely new population groups, and this breaks down the entire study.) I’m also not entirely sure that using saturation as a guide would work, because saturation occurs when no new information is forthcoming. What if a clinical test has been ordered, but the results are not yet in. With the current clinical examinations of symptoms having being completed on the patient, if no new information has been gathered, then saturation has been reached before the results of those tests are to be viewed. Grounded theory then demands that those test results be not viewed, and be ignored. In fact, phone the lab and cancel the test. Would any clinician really want to do that? Note that, if not all questions have been answered, or if there is an unresolved contradiction, GT does not require further sampling – saturation has been reached based on no new information. But in clinical reasoning, surely the results of that test may resolve a contradiction, or indeed, raise a whole new set of questions. As I say, a brave attempt at yoking with some really interesting ideas being generated, but I’m not entirely convinced that it would work. It might be useful if the authors could actually test it with real cases, and modify it as necessary.

Reviewer Expertise:

NA

No decision status is available

# Reviewer report: Reviewer response for version 1

**DOI:** 10.21956/mep.19413.r28495

**Published:** 2017-01-05

**Authors:** Trevor Gibbs

**Affiliations:** 1 AMEE

This review has been migrated. The reviewer awarded 3 stars out of 5 An interesting paper that covers an interesting but difficult topic. Any paper that tries to bring clarity to the teaching of clinical reasoning is always worthwhile reading, for the reasons explored and discussed by the authors at the beginning of this paper.The authors draw a novel parallel between the clinical reasoning process and grounded theory- connecting the process to the various stages of using grounded theory during research., and it was enlightening to read these parallels. However, after reading the paper I was still at a loss to see how drawing these parallels improved the teaching of clinical reasoning. The stages of clinical reasoning are well highlighted in the press, it is being able to put these together in a logical teaching progression that seems to be the problem.

Reviewer Expertise:

NA

No decision status is available
